# Giant right coronary artery aneurysms presenting as a cardiac mass: Case report

**DOI:** 10.1097/MD.0000000000004924

**Published:** 2016-09-23

**Authors:** Huanhuan Wang, Yin Zhang, Yanbo Xie, Hongyue Wang, Jinqing Yuan

**Affiliations:** a Division of Cardiology; b Division of Cardiovascular Surgery; c Division of Pathology, Fuwai Hospital, National Center for Cardiovascular Disease, Beijing, China.

**Keywords:** coronary angiography, coronary artery aneurysm, coronary computed tomography angiography

## Abstract

**Introduction:**

Coronary artery aneurysm (CAA) is defined as coronary dilatation which exceeds the diameter of the normal adjacent artery segments or the diameter of the patient's largest coronary artery by 1.5 times. The incidence of giant CAA is difficult to be determined, since only few reports have been described in the literature.

**Methods and Results:**

A 65-year-old man was referred to our hospital because of a “mass” in the right heart detected on echocardiography at a regular medical health examination, while he experienced no any symptoms. Coronary angiography showed the severe stenosis of the left anterior descending artery (LAD) and the left circumflex artery (LCX) and the diffusely ectatic change of the right coronary artery (RCA), but no mass was found in any of these arteries. Coronary computed tomography angiography (CTA) confirmed that the “mass” was the giant aneurysms of RCA with thrombus. He received coronary artery bypass graft (CABG) with thrombectomy. The histopathology showed the deposits of lipid and hyalin in the tunica intima, the focal calcifications, the very thin tunica media, and the disappearance of the part of the tunica media in the RCA.

**Conclusions:**

Coronary artery aneurysm which may contain thrombus can complicate a diagnostic coronary angiography due to the risk of distal embolization and may lead to myocardial infarction. This case report demonstrates 2 RCA aneurysms with a thrombus presenting as a giant “mass” which was successfully treated by CABG with thrombectomy.

## Abstract 2

Supplemental Digital Content is available in the text

## 1 Introduction

Coronary artery aneurysm (CAA) is defined as coronary dilatation which exceeds the diameter of the normal adjacent artery segments or the diameter of the patient's largest coronary artery by 1.5 times. The “giant” CAA definition is still controversial. Some authors consider coronary aneurysms to be giants when the CAA is >2 cm, whereas the Committee of the American Heart Association has defined giant aneurysms as those >8 mm.^[1,2]^ The “giant” CAA is a rare diagnosis.^[3]^

The presence of CAA was first recognized in post-mortem studies. It was initially thought that Bougon described the first case in 1812.^[4]^ However, it turns out that the first pathologic description of CAA was by Giovanni Battista Morgagni in 1761.^[5]^ The first case series of 31 cases was reported in 1929.^[6]^ However, CAA was first diagnosed in a live patient by coronary angiography by Munker in 1958.^[7]^ CAA is a rare condition. The incidence of coronary aneurysm varies widely in different studies. It was reported to be 1.4%,^[8]^ 4.9%,^[9]^ or between 1.5% and 5%.^[7]^ The order of the most affected coronary artery is RCA, LAD, LCX, and left main (LM) that is rarely affected.^[10]^ CAA is frequently asymptomatic, so it is generally an incidental or coincidental finding on coronary angiography. In symptomatic cases, it is usually caused by myocardial ischemia. The incidence of giant CAA is difficult to be determined, since only few giant CAA cases have been described in the literature.^[11]^

## 2 Case presentation

**Figure 1:**
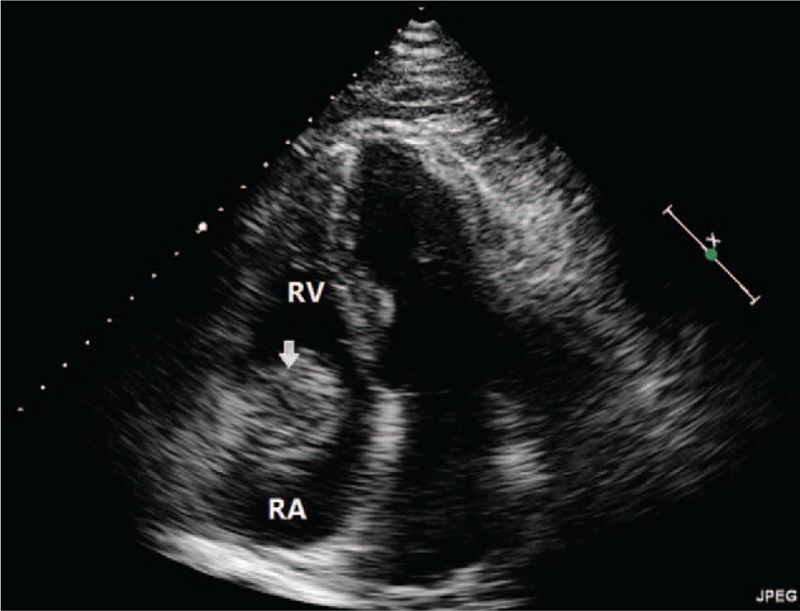
Transthoracic echocardiography revealed a 37 × 37 mm^2^ round like acoustic shadow above the tricuspid valve ring and showed no swing in the chambers of the heart. The internal echogenicity was medium mixed with hypoechoic signal with a strong echo capsule and a clear boundary.

**Figure 2:**
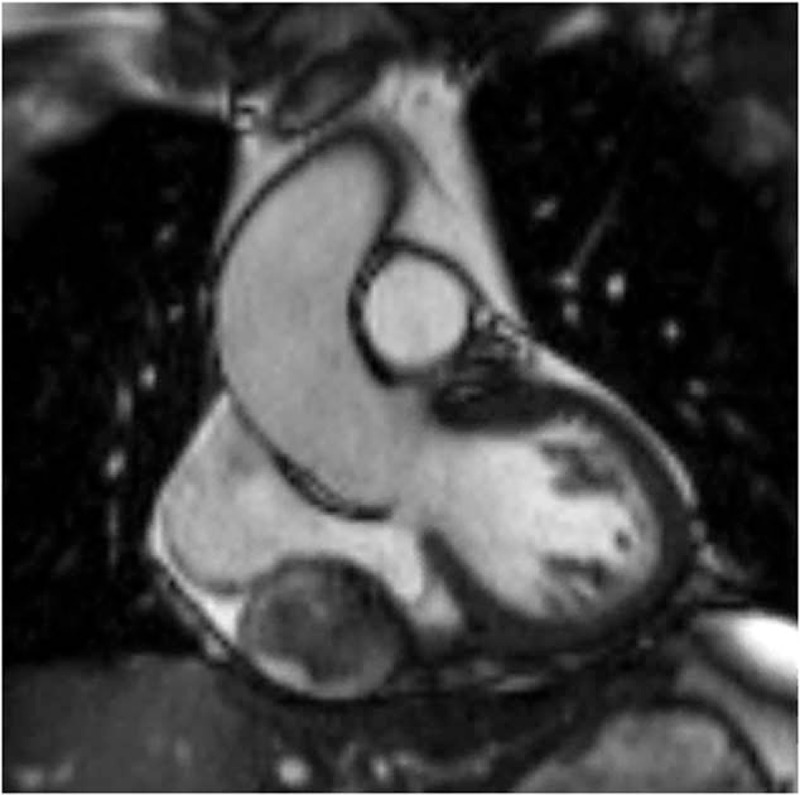
Magnetic resonance imaging depicted a tubular shadow, which compressed the right atrium, under the pericardium adjacent to the right atrioventricular groove, with an internal heterogeneous acoustic signal and smooth edges. Parts of the shadow and the wall showed a delayed enhancement.

**Figure 3:**
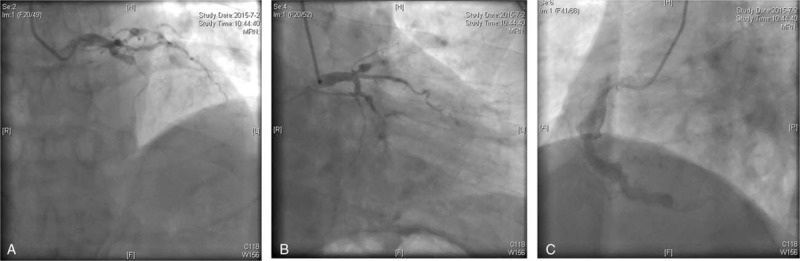
Invasive coronary angiography showed an ectasia in the proximal LAD and the total occlusion in the mid LAD (A). The LCX had diffuse 80–90% stenosis from the mid to the distal end (B). The RCA had diffusely ectactic change (C,D). LAD = left anterior descending artery, RCA = right coronary artery.

**Figure 4:**
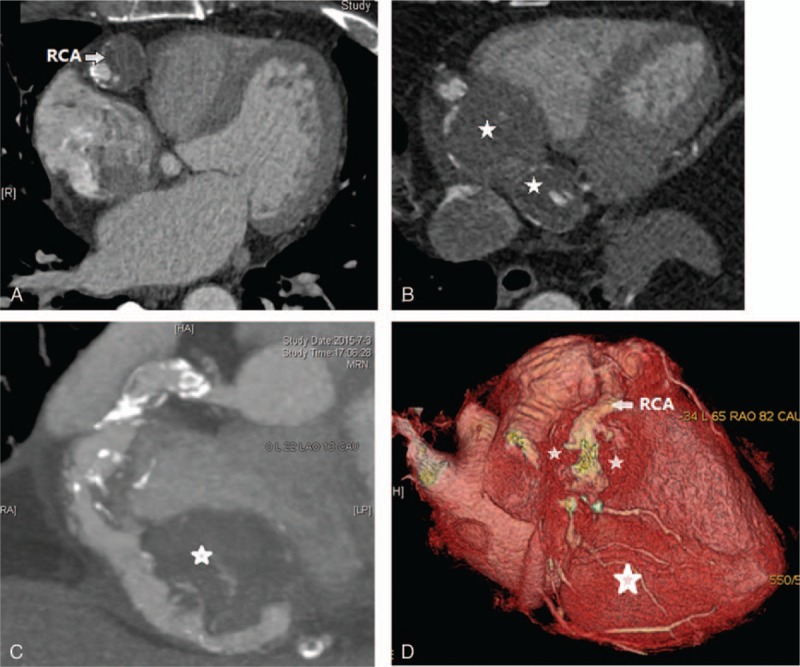
Coronary CTA confirmed that the “mass” were the giant aneurysms of RCA with thrombosis (A, B). The maximum diameters were 25 and 38 mm, respectively, which caused the compression of the right atrium (C). CTA = computed tomography angiography, RCA = right coronary artery.

**Figure 5:**
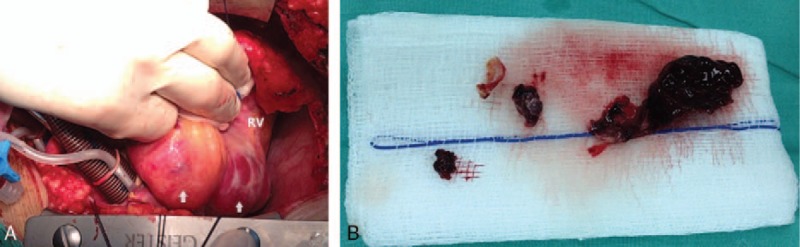
Intraoperatively, 2 adjacent aneurysms were seen in the mid and the distal RCA, compressing the right atrium (A) and a massive intraluminal thrombus was confirmed and removed from the RCA aneurysms (B). RCA = right coronary artery.

**Figure 6:**
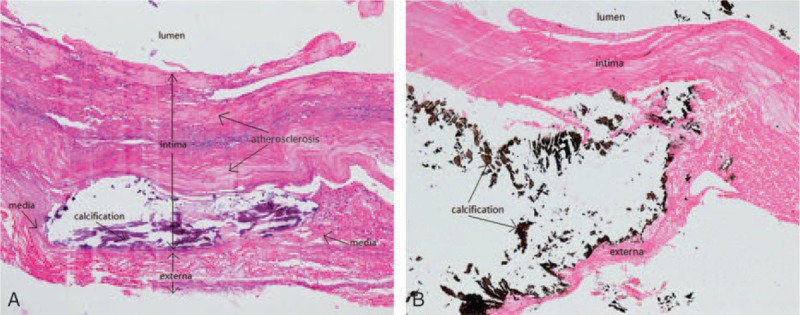
The histopathological section of the RCA aneurysm showed the presence of atherosclerosis (A) with calcification (B), a very thin tunica media and the partial disappearance of the tunica media. RCA = right coronary artery.

A 65-year-old man was referred to our hospital because of a “mass” in the right heart detected on echocardiography at a regular medical health examination. He did not experience angina-like chest pain or any other symptoms. He had no history of Kawasaki disease or chest trauma. On physical examination, his blood pressure was 110/70 mm Hg and pulse rate was 61/min. Precordial auscultation disclosed no abnormalities. Physical examination results were normal. The blood tests associated with the immune system were normal. The chest roentgenogram was normal. Electrocardiogram was normal without Q waves. However, transthoracic echocardiography revealed a 37 × 37 mm^2^ round like acoustic shadow above the tricuspid valve ring and showed no swing in the chambers of the heart. The internal echogenicity was medium mixed with hypoechoic signal and a strong echo capsule and clear boundary (Fig. 1, Supplemental Digital Content UCG 1, 2). Magnetic resonance imaging (MRI) depicted a tubular shadow under the pericardium adjacent to the right atrioventricular groove. This shadow that appeared to compress the right atrium had an internal heterogeneous acoustic signal and smooth edges. The parts of the shadow and the wall showed a delayed enhancement (Fig. 2, Supplemental Digital Content MRI 1, 2). The invasive coronary angiography showed an ectasia in the proximal LAD, a total occlusion in the mid LAD (Fig. 3A), a 80–90% diffuse stenosis from the mid to distal end of the LCX (Fig. 3B), and a diffuse ectatic change of the RCA (Fig. 3C, Supplemental Digital Content CAG1, 2, 3, 4). However, no any sign of cardiac mass was found on angiogram. In order to further confirm the location of the cardiac “mass” and whether the cardiac “mass” detected by the transthoracic echocardiography and MRI was a CAA, coronary CTA was performed on this patient. Coronary CTA confirmed that the “mass” was the giant aneurysms of RCA with thrombosis (Fig. 4A and B). The maximum diameters of these giant aneurysms were 25 and 38 mm, respectively. The mass caused the compression of the right atrium (Fig. 4C and D). Furthermore, SPECT was performed for the evidence of ischemia, which showed a reversible intensity defect in the apical, septal, and anterior wall and irreversible defect in sidewall near the apex of the left ventricle, suggestive of ischemia in the distribution of LAD. Therefore, CABG was recommended considering the risk of rupture, ischemia, and thromboembolism. He received bypass grafts from the left internal mammary artery to LAD and the saphenous vein grafts to the posterior descending artery, the posterior lateral artery, and the obtuse marginal artery. Intraoperatively, 2 adjacent aneurysms were found in the mid and distal RCA, compressing the right atrium (Fig. 5A, Supplemental Digital Content CABG). In addition, the presence of a massive intraluminal thrombus detected by coronary computed tomography in the RCA aneurysms was confirmed and it was removed from the RCA aneurysms (Fig. 5B). The histopathology showed the deposits of lipid and hyalin in the tunica intima (Fig. 6A), the presence of focal calcifications (Fig. 6B, Von Kossa's stain), and a very thin tunica media and the disappearance of the part of the tunica media in the RCA. He was discharged 10 days later after the surgery. Upon discharge, he was prescribed with aspirin 100 mg and warfarin 3 mg daily with the International Normalized Ratio between 2 and 3 and without any adverse cardiac event.

## 3 Discussion

The causes of CAA can be occasionally congenital or mainly acquired. The congenital CAA is generally found in young patients.^[12,13]^ The main acquired etiological factor for the development of CAA is atherosclerotic coronary artery disease, which is present in 50% to 52% of cases. The histologic features of an atherosclerotic aneurysm include lipid and hyalin deposits in the intima, disruption of intima and media, focal calcification and fibrosis, cholesterol crystals, thrombi, and inflammatory cells.^[14]^ Other acquired causes include trauma, connective tissue disorders, and inflammatory disorders such as Marfan syndrome, Ehlers Danlos syndrome, Kawasaki disease, Takayasu arteritis, and Behcet's disease.^[15]^ In our case, the histopathology showed the presence of lipid and hyalin deposits in the tunica intima, focal calcifications, and the thinning and the partial disappearance of the tunica media in the RCA. All these pieces of evidence support that the cause of CAA in our case is also atherosclerosis.

The CAA can generally be detected by coronary angiography. Transthoracic echocardiography and MRI suggested that our case had a cardiac mass. However, coronary angiography failed to detect the 2 giant CAAs that were eventually confirmed by coronary CTA. When an aneurysm is filled with thrombosis, it cannot be seen on coronary angiography. This may explain why coronary angiography failed to detect the 2 giant CAAs with thrombosis in our case.

CAA occurs more frequently in males than in females.^[6,16]^ In contrast, Toshiaki et al ^[17]^ reviewed the reported cases of the giant CAA with a diameter of 50 mm or more without fistula in the literature and they found no sex predomination for giant CAA. Although our case was a male patient who had 2 adjacent giant CAAs, it remains unknown whether giant CAA truly occurs without a gender preference.

The most affected coronary artery is the RCA.^[10]^ Our case had 2 aneurysms with thrombus in the RCA, which is in agreement with the literature. Patients with CAA are generally symptomatic. For those patients who experience CAA-related symptoms, the most common symptoms are the chest pain, dyspnea, and palpitation. Although our case had 2 adjacent giant CAAs, he did not experience any symptoms, suggesting that a patient with giant CAAs can still be asymptomatic and if it is not an incidental finding like in our case, it is still hard to detect CAAs and even giant CAAs in patients without any symptoms.

The management of CAA consists of medical management, stent insertion, and surgical excision.^[18]^ Surgery has been recommended for all CAA in view of the risk of thrombosis or rupture, particularly for giant aneurysm. Surgical intervention for giant CAA appears to be the treatment of choice in the reported literature.^[19]^ Resection of coronary artery aneurysm with CABG is the most frequently chosen treatment modality for giant CAA.^[20]^ Covered stents were also reported to treat giant CAA.^[21–23]^ Antiplatelet and anticoagulation therapy with a regular follow-up and percutaneous coronary intervention with possible stenting are available options. However, the best treatment strategy for CAA is still controversial, as there is no single management strategy that treats all CAA equally well. Therefore, it is recommended that patients be managed individually according to the location of the aneurysm and the clinical context.^[24]^ In the present case, the coronary angiography showed that RCA had diffusely ectatic change without a “mass” or thrombus. However, coronary CTA showed that RCA had 2 giant CAAs with thrombus, suggesting that the patient had the evidence of ischemia and was at the risk of further thromboembolism. Taken together, CABG with thrombectomy was performed rather than resection of the CAAs. The histopathological evidence also supports the choice of treatment for this case.

## 4 Conclusion

Coronary artery aneurysm which may contain thrombus can complicate a diagnostic coronary angiography due to the risk of distal embolization and may lead to myocardial infarction. This case report demonstrates 2 RCA aneurysms with a thrombus presenting as a giant “mass,” which was successfully treated by CABG with thrombectomy.

## Supplementary Material

Supplemental Digital Content

## Supplementary Material

Supplemental Digital Content

## Supplementary Material

Supplemental Digital Content

## Supplementary Material

Supplemental Digital Content

## Supplementary Material

Supplemental Digital Content

## Supplementary Material

Supplemental Digital Content

## Supplementary Material

Supplemental Digital Content

## Supplementary Material

Supplemental Digital Content

## Supplementary Material

Supplemental Digital Content
